# Association between serum calcium levels and uterine leiomyoma: a retrospective case–control study exploring the potential mediating roles of inflammatory and lipid-related indicators

**DOI:** 10.3389/fnut.2026.1871649

**Published:** 2026-07-22

**Authors:** Yanan Duan, Xiaotong Cui, Fanmao Kong, Huaqin Sun, Aiping Chen, Yushuang Yao

**Affiliations:** 1 Department of Gynecology, The Affiliated Hospital of Qingdao University, Qingdao, Shandong, China; 2 Qingdao Medical College, Qingdao University, Qingdao, Shandong, China; 3 Zhongshan Road Community Health Service Center of Shinan District, Qingdao, Shandong, China

**Keywords:** inflammation, lipid metabolism, mediation analysis, serum calcium, uterine leiomyoma

## Abstract

**Background:**

The occurrence of uterine leiomyoma (UL) is closely related to metabolic abnormalities and inflammatory reactions, but the role of serum calcium in it and its relationship with inflammation and lipid metabolism are not yet clear. The aim of this study is to explore the association between serum calcium and UL risk, and evaluate the mediating role of inflammation and lipid indicators in it.

**Method:**

This study included 1,169 UL patients and 985 controls, and propensity score matching (PSM) was used to reduce confounding bias. Using logistic regression analysis to evaluate the association between serum calcium and UL, and combining generalized additive modeling (GAM) to explore nonlinear relationships and threshold effects. Meanwhile, the mediating role of inflammation and lipid indicators in this association was quantified through bootstrap mediation analysis.

**Result:**

Serum calcium was significantly negatively correlated with UL risk and remained stable after PSM correction. Nonlinear analysis shows a significant threshold effect between it and UL. In the mediation analysis, some inflammatory and lipid indicators play a significant mediating role between serum calcium and UL, suggesting that the metabolic inflammatory pathway is involved in the occurrence of UL.

**Conclusion:**

Serum calcium levels were associated with UL risk, and several inflammatory and lipid-related indicators showed significant statistical mediation effects in this association. The relevant indicators are derived from routine testing and have potential clinical application value, but further research is needed to verify their causal relationship.

## Background

Uterine leiomyoma (UL) are one of the more common types of benign tumors in women of childbearing age, with a prevalence rate of 20–40%, and are closely related to clinical manifestations such as abnormal uterine bleeding, infertility, and chronic pelvic pain (1–3). Although it occurs frequently in the population and has a certain impact on women’s health, the specific biological processes underlying the occurrence and progression of UL have not been fully elucidated. Current research suggests that changes in metabolic status and immune inflammatory responses may be involved, but the relevant modes of action and their interrelationships have not yet formed a consensus (4, 5).

In recent years, research has gradually noticed that changes in metabolic status are often accompanied by sustained low-grade inflammatory reactions, which have also been observed in UL related populations (6, 7). In various gynecological diseases, there is often a synchronous change between fluctuations in blood lipid levels and inflammatory responses (8, 9). However, most studies mainly focus on statistical associations, and there is still a lack of clear descriptions of how these metabolic factors affect the occurrence of UL through the inflammatory process and the related pathways. In this context, serum calcium, as an important indicator of mineral metabolism, has been widely reported for its involvement in cell proliferation, apoptosis, and signal transduction. Previous observations suggest that changes in calcium ion levels may affect the growth status of smooth muscle cells and are also related to changes in the inflammatory environment (10, 11). However, the specific manifestations and interrelationships of these phenomena in UL still require further analysis. Studies have suggested that metabolic abnormalities may be accompanied by a sustained increase in inflammatory response, providing conditions for abnormal proliferation of uterine smooth muscle cells (12). In this process, the mutual influence between lipid changes and inflammatory responses may constitute an explanatory pathway linking metabolic changes with disease occurrence.

In the above context, this study used serum calcium as the main observed variable and included inflammation and lipid related indicators in the analysis framework to evaluate their possible roles in the occurrence of UL. The study adopted a case–control design and introduced Propensity Score Matching (PSM) to improve baseline differences between groups, thereby reducing the influence of confounding factors to some extent. In statistical analysis, the relationship between serum calcium and UL risk is further explored by combining generalized additive models (GAM) and segmented regression methods to identify possible nonlinear trends and their corresponding intervals. Meanwhile, through mediation analysis, the roles of inflammation and lipid indicators in this association were quantitatively evaluated. Based on the above analysis, this study attempts to explain the occurrence process of UL from the perspective of the interrelationship between serum calcium, inflammatory response, and lipid changes, and provide reference for subsequent risk assessment and related interventions.

## Method

### Study population and design

**Figure 1:**
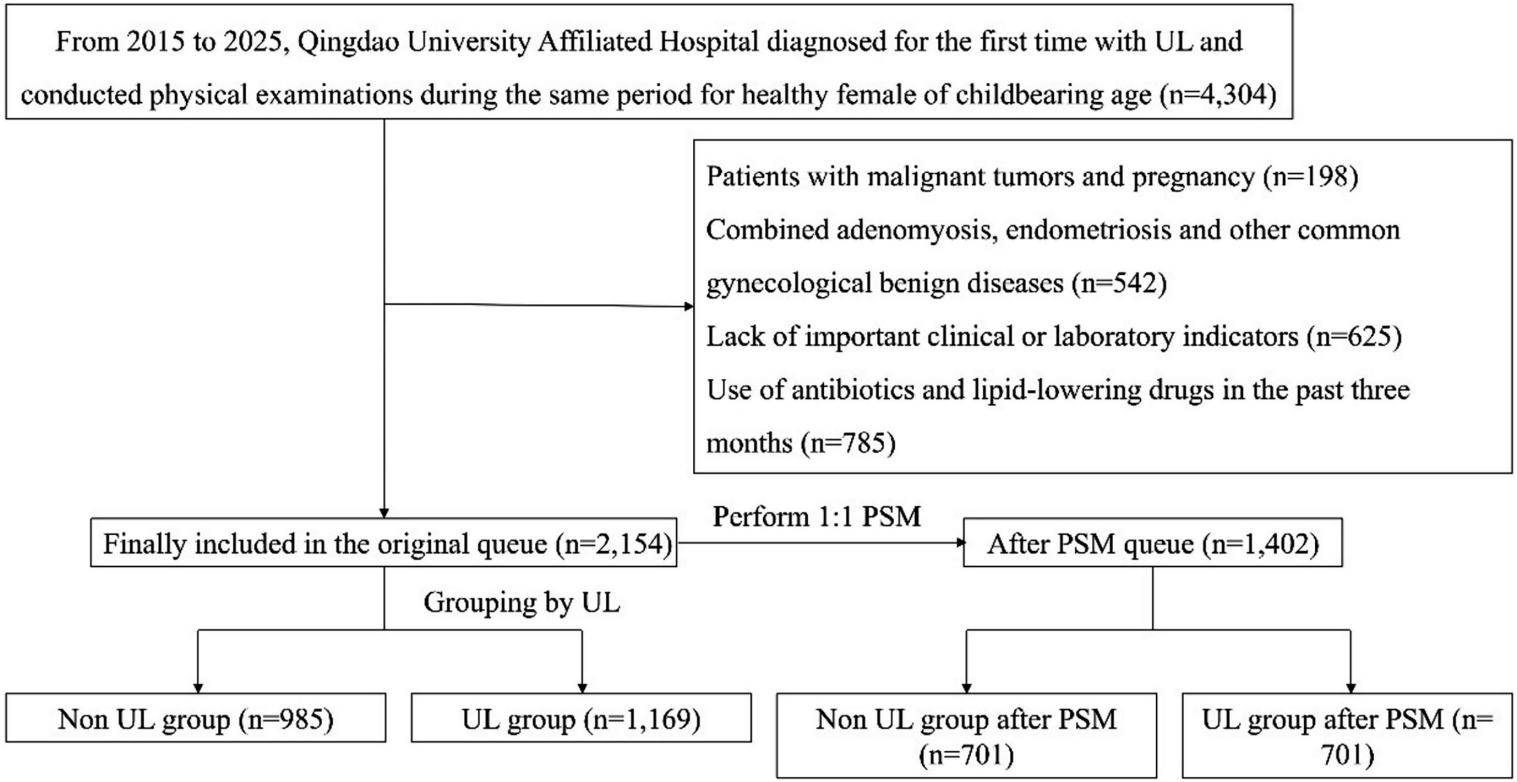
Acceptance and arrangement process. PSM, propensity score matching; UL, uterine leiomyoma.

This study adopted a retrospective case–control design, and relevant data were extracted from the clinical database of Qingdao University Affiliated Hospital from January 2015 to 2025. The sample size was determined by the number of eligible participants available in the hospital database during the predefined study period. Because this was a retrospective case–control study based on existing data, no *a priori* power calculation was performed. The study included female patients diagnosed with UL for the first time during this period, as well as women of childbearing age who underwent physical examinations during the same period, with a total sample size of 4,304. In the process of data organization, samples are screened based on established inclusion and exclusion criteria. Patients with malignant tumors or pregnancy (*n* = 198), adenomyosis, endometriosis, and other common benign gynecological diseases (*n* = 542), missing important clinical or laboratory indicators (*n* = 625), or recent use of antibiotics and lipid-lowering drugs within the previous 3 months (*n* = 785) were excluded. The detailed participant selection process is presented in Figure 1. After the above screening, 2,154 individuals were finally included in the subsequent analysis queue.

Based on whether diagnosed with UL, the study subjects were divided into a case group and a control group, including 1,169 cases and 985 cases, respectively. Considering that there may be differences in baseline features between the two groups, a 1:1 PSM method is further introduced to adjust the samples to reduce the influence of confounding factors. After matching, a total of 1,402 individuals were included in the analysis, with 701 in the UL group and 701 in the control group (Figure 1). This research process complies with the relevant requirements of the Helsinki Declaration and has been approved by the hospital ethics committee.

### Variables

This study used UL as the outcome variable. Serum calcium is used as the primary exposure variable to evaluate its relationship with the risk of UL occurrence. Covariance refers to demographic characteristics and clinical data, including age, height, weight, body mass index, overweight status, medical history, surgical history, smoking history, and alcohol consumption history. History of past illnesses was defined as the presence of any previously documented chronic medical condition recorded in the electronic medical records. This variable was analyzed as a binary indicator (yes/no). The above information was collected by trained medical personnel through standardized questionnaires during admission or physical examination. Among them, overweight status, medical history, surgical history, smoking and alcohol consumption were all treated as categorical variables.

The laboratory indicators include inflammation related indicators and blood lipid indicators. All laboratory tests are completed in the hospital’s central laboratory, using standardized automatic analysis equipment and strictly implementing indoor and inter room quality control. All blood samples were collected on an empty stomach and tested within 1 h after collection to ensure the accuracy and reliability of the results. Inflammatory indicators include platelet count (PLT), lymphocyte count (LBC), neutrophil count (NEU). Blood lipid indicators include high-density lipoprotein (HDL), low-density lipoprotein (LDL), total cholesterol (TC), and triglycerides (TG). The relevant derivative indicators are calculated according to the following formula.

Body Mass Index (BMI) = Weight (kg)/Height (m)^2^

Platelet to lymphocyte ratio (PLR) = PLT/LBC

Neutrophil to lymphocyte ratio (NLR) = NEU/LBC

Mononuclear to lymphocyte ratio (MLR) = MBC/LBC

Systemic immune inflammatory index (SII) = PLT*NEU/LBC

White to globulin ratio (A/G) = albumin/globulin

Non high density lipoprotein to high density lipoprotein ratio (NHHR) = (TC-HDL)/HDL

### Statistical analysis

All statistical analyses were conducted using R software^1^ Completed. Continuous variables are represented by mean ± standard deviation (SD), while categorical variables are represented by frequency and percentage. To reduce the influence of potential confounding factors, this study used a 1:1 nearest neighbor matching method for PSM, without replacement matching, and set the caliper value to 0.02. The covariates included in the matching include age, height, weight, BMI, past medical history (cardiovascular disease, diabetes, tumor), surgical history, smoking and drinking, etc. By evaluating the inter group balance before and after matching through standardized mean differences (SMDs), if SMD < 0.1, it is considered to have achieved good balance. Firstly, single factor logistic regression analysis was used to screen potential factors related to UL, and variables with statistical significance were included in the multi factor logistic regression model. The odds ratio (OR) and its 95% confidence interval (CI) were calculated. Because serum calcium is physiologically maintained within a narrow range, serum calcium was rescaled and modeled per 0.1 mmol/L increase to improve the clinical interpretability of the estimated effect sizes. Accordingly, the reported ORs for serum calcium represent the change in UL odds associated with each 0.1 mmol/L increase in serum calcium concentration. Build multiple models and gradually adjust potential confounding factors to evaluate the independent associations between variables and UL. To explore the nonlinear relationship between continuous variables and UL risk, a generalized additive model (GAM) was used for fitting and a smooth fitting curve was plotted. When there is a non-linear relationship, a two-stage linear regression model is further used to identify potential inflection points (thresholds), and the optimal threshold is determined through maximum likelihood estimation, while likelihood ratio testing is used for model comparison. On this basis, the variables are segmented based on the identified thresholds, and threshold effect analysis is conducted to estimate the magnitude of effects in different intervals. To explore potential biological mechanisms, this study used mediation analysis to evaluate the statistical mediation effects of inflammation and lipid indicators in the association between serum calcium and UL. Mediation analyses were conducted using a counterfactual causal mediation framework. Because UL status was a binary outcome, generalized linear models with a probit link function were used for the outcome model. Total effects, average causal mediation effects, average direct effects, and corresponding bootstrap confidence intervals were estimated using 1,000 nonparametric bootstrap resamples. When the indirect effect has statistical significance, it is considered that there is a significant mediating effect. In addition, a multiple factor linear regression model was constructed to analyze the correlation between serum calcium and inflammation and lipid indicators, and to adjust for relevant confounding factors. All statistical tests are two-sided tests, with *p* < 0.05 indicating statistically significant differences.

## Result

### Baseline characteristics and association analysis

This study included a total of 2,154 research subjects, including 1,169 in the UL group and 985 in the control group. The specific inclusion process is shown in Figure 1. Before PSM, there were significant differences in demographic characteristics and multiple clinical indicators between the two groups, including height, previous medical history, hypertension, coronary heart disease, diabetes, smoking and drinking, as well as multiple inflammatory and lipid indicators (all *p* < 0.05) (Supplementary Table 1), indicating that there was significant imbalance at baseline. To reduce the influence of confounding factors, this study used a 1:1 PSM method, and finally included 1,402 subjects (701 in the UL group and 701 in the control group) after matching. After PSM, the baseline characteristics of the two groups became substantially more balanced. Although small residual differences remained for age (SMD = 0.154), height (SMD = −0.123), and history of past illnesses (SMD = 0.120), all other covariates achieved SMD values below 0.1, indicating generally acceptable covariate balance after matching. To further reduce the potential influence of these residual imbalances, age, height, and history of past illnesses were included as covariates in the multivariable regression analyses (Model IV). In the matched population, there were still significant differences between the UL group and the control group in multiple biochemical indicators (Table 1). Specifically, the serum calcium levels in the UL group were significantly reduced (2.27 ± 0.14 vs. 2.31 ± 0.15 mmol/L, *p* < 0.001), while WBC, MBC, and HB were lower, while PLT, PLR, and SII were significantly increased (all *p* < 0.05). In addition, albumin and A/G ratio increased, while TC, TG, LDL, and NHHR showed varying degrees of decrease in the UL group (Table 1).

**Table 1:** Baseline feature description of the study population after PSM.

Variable	Total (*n* = 1,402) Mean±SD/*N* (%)	No-UL (*n* = 701) Mean±SD/*N* (%)	UL (*n* = 701) Mean±SD/*N* (%)	*p*-value	SMD
Age, years	43.91 ± 9.28	43.40 ± 11.33	44.42 ± 6.58	0.041	0.154
Weight, kg	64.32 ± 10.19	64.45 ± 10.78	64.20 ± 9.56	0.651	−0.026
Height, cm	161.28 ± 5.03	161.62 ± 4.49	160.94 ± 5.50	0.012	−0.123
BMI, kg/m^2^	24.73 ± 3.83	24.66 ± 3.91	24.81 ± 3.75	0.475	0.039
History of past illnesses, *n* (%)				0.021	
No	999 (71.26%)	519 (74.04%)	480 (68.47%)		−0.120
Yes	403 (28.74%)	182 (25.96%)	221 (31.53%)		0.120
History of hypertension, *n* (%)				0.406	
No	1,302 (92.87%)	655 (93.44%)	647 (92.30%)		−0.043
Yes	100 (7.13%)	46 (6.56%)	54 (7.70%)		0.043
History of CHD, *n* (%)				1.000	
No	1,392 (99.29%)	696 (99.29%)	696 (99.29%)		0.000
Yes	10 (0.71%)	5 (0.71%)	5 (0.71%)		0.000
History of diabetes, *n* (%)				0.884	
No	1,353 (96.50%)	676 (96.43%)	677 (96.58%)		0.008
Yes	49 (3.50%)	25 (3.57%)	24 (3.42%)		−0.008
History of previous surgeries, *n* (%)				0.075	
No	1,008 (71.90%)	489 (69.76%)	519 (74.04%)		0.098
Yes	394 (28.10%)	212 (30.24%)	182 (25.96%)		−0.098
Smoking, *n* (%)				1.000	
No	1,394 (99.43%)	697 (99.43%)	697 (99.43%)		0.000
Yes	8 (0.57%)	4 (0.57%)	4 (0.57%)		0.000
Alcoholism, *n* (%)				0.705	
No	1,395 (99.50%)	697 (99.43%)	698 (99.57%)		0.022
Yes	7 (0.50%)	4 (0.57%)	3 (0.43%)		−0.022
Serum calcium, mmol/L	2.29 ± 0.14	2.31 ± 0.15	2.27 ± 0.14	<0.001	–
WBC, 10^9^/L	6.04 ± 2.27	6.24 ± 2.63	5.84 ± 1.83	0.001	–
RBC, 10^12^/L	4.36 ± 0.44	4.32 ± 0.41	4.40 ± 0.47	0.002	–
LBC, 10^9^/L	1.86 ± 1.15	1.92 ± 1.54	1.80 ± 0.54	0.040	–
MBC, 10^9^/L	0.38 ± 0.16	0.40 ± 0.17	0.37 ± 0.14	<0.001	–
NEU, 10^9^/L	3.68 ± 1.79	3.80 ± 1.94	3.57 ± 1.61	0.016	–
HB, g/L	121.47 ± 22.20	126.49 ± 17.10	116.46 ± 25.37	<0.001	–
PLT, 10^9^/L	256.01 ± 75.47	232.25 ± 65.74	279.78 ± 77.12	<0.001	–
PLR	154.23 ± 74.20	138.04 ± 68.41	170.42 ± 76.25	<0.001	–
NLR	2.27 ± 2.16	2.33 ± 1.95	2.22 ± 2.35	0.337	–
MLR	0.23 ± 0.13	0.23 ± 0.14	0.22 ± 0.12	0.041	–
SII	578.69 ± 484.84	544.03 ± 479.84	613.36 ± 487.67	0.007	–
Albumin, g/L	45.94 ± 7.34	42.99 ± 6.48	48.79 ± 6.98	<0.001	–
Globulin, g/L	28.97 ± 4.23	29.53 ± 4.48	28.42 ± 3.91	<0.001	–
A/G	1.51 ± 0.29	1.44 ± 0.30	1.57 ± 0.26	<0.001	–
TC, mmol/L	5.08 ± 1.13	5.29 ± 1.26	4.89 ± 0.96	<0.001	–
TG, mmol/L	1.23 ± 0.96	1.38 ± 1.10	1.10 ± 0.80	<0.001	–
HDL, mmol/L	1.54 ± 0.37	1.54 ± 0.41	1.54 ± 0.34	0.911	–
LDL, mmol/L	2.82 ± 0.87	2.87 ± 0.84	2.77 ± 0.90	0.031	–
NHHR	2.45 ± 1.28	2.63 ± 1.63	2.28 ± 0.81	<0.001	–

PSM, propensity score matching; UL, uterine leiomyoma; SD, standard deviation; SMD, standardized mean difference; BMI, body Mass Index; CHD, coronary heart disease; WBC, white blood cell count; RBC, red blood cell count; LBC, lymphocyte count; MBC, monocyte count; NEU, neutrophil count; HB, hemoglobin; PLT, platelet count; PLR, platelet-to-lymphocyte ratio; NLR, neutrophil-to-lymphocyte ratio; MLR, monocyte-to-lymphocyte ratio; SII, systemic immune-inflammation index; A/G, albumin-to-globulin ratio; TC, total cholesterol; TG, triglycerides; HDL, high-density lipoprotein cholesterol; LDL, low-density lipoprotein cholesterol; NHHR, non-HDL cholesterol to HDL cholesterol ratio.

Univariate logistic regression analysis showed that serum calcium was significantly and inversely associated with UL both before and after PSM. When serum calcium was modeled per 0.1 mmol/L increase, the ORs were 0.82 (95% CI: 0.77–0.87, *p* < 0.001) before PSM and 0.83 (95% CI: 0.77–0.90, *p* < 0.001) after PSM, indicating that each 0.1 mmol/L increase in serum calcium was associated with approximately 17–18% lower odds of UL. In terms of inflammation and blood lipid indicators, WBC, LBC, MBC, HB, TC, TG, LDL, and NHHR are also significantly correlated with UL, while PLR, SII, and other indicators are shown as risk factors (Supplementary Table 2). Further multivariable logistic regression analyses demonstrated that the inverse association between serum calcium and UL remained significant after adjustment for demographic characteristics, lifestyle factors, and comorbidities. In the fully adjusted model after PSM (Model IV), each 0.1 mmol/L increase in serum calcium was associated with 17% lower odds of UL (OR = 0.83, 95% CI: 0.77–0.90, *p* < 0.001). Similar findings were observed before PSM, supporting the robustness of the association. Meanwhile, WBC, MLR, TC, TG, LDL, and NHHR remained significant correlated factors, while PLR, SII, albumin, and A/G ratio were positively correlated with UL (Table 2). It is worth noting that in the multivariate analysis before PSM, the direction and significance of the above variables were generally consistent (Supplementary Table 3), indicating that the research results have good stability. In addition, the multiple regression analysis between serum calcium and inflammation and lipid indicators showed similar trends before and after PSM (Supplementary Tables 4, 5), further supporting the robustness of the results.

**Table 2:** UL multiple regression analysis after PSM.

Exposure	Model I OR (95% CI) *p*-value	Model II OR (95% CI) *p*-value	Model III OR (95% CI) *p*-value	Model IV OR (95% CI) *p*-value
Serum calcium (per 0.1 mmol/L increase)	0.83 (0.77, 0.90) < 0.0001	0.83 (0.77, 0.89) < 0.0001	0.84 (0.78, 0.91) < 0.0001	0.83 (0.77, 0.90) < 0.0001
WBC	0.92 (0.87, 0.97) 0.0013	0.92 (0.87, 0.97) 0.0030	0.92 (0.87, 0.97) 0.0014	0.92 (0.87, 0.97) 0.0036
PLR	1.01 (1.01, 1.01) < 0.0001	1.01 (1.01, 1.01) < 0.0001	1.01 (1.01, 1.01) < 0.0001	1.01 (1.01, 1.01) < 0.0001
NLR	0.98 (0.93, 1.03) 0.3478	0.98 (0.93, 1.03) 0.4370	0.97 (0.92, 1.03) 0.3362	0.98 (0.93, 1.03) 0.4260
MLR	0.42 (0.18, 0.98) 0.0442	0.44 (0.19, 1.04) 0.0622	0.40 (0.17, 0.94) 0.0353	0.42 (0.18, 1.00) 0.0508
SII	1.00 (1.00, 1.00) 0.0092	1.00 (1.00, 1.00) 0.0058	1.00 (1.00, 1.00) 0.0095	1.00 (1.00, 1.00) 0.0056
Albumin	1.14 (1.12, 1.16) < 0.0001	1.14 (1.12, 1.16) < 0.0001	1.14 (1.12, 1.16) < 0.0001	1.14 (1.12, 1.16) < 0.0001
Globulin	0.94 (0.91, 0.96) < 0.0001	0.94 (0.91, 0.96) < 0.0001	0.94 (0.91, 0.96) < 0.0001	0.94 (0.91, 0.96) < 0.0001
A/G	6.00 (3.91, 9.21) < 0.0001	6.46 (4.17, 10.00) < 0.0001	6.15 (3.99, 9.48) < 0.0001	6.64 (4.27, 10.33) < 0.0001
TC	0.71 (0.64, 0.79) < 0.0001	0.68 (0.61, 0.76) < 0.0001	0.71 (0.64, 0.79) < 0.0001	0.68 (0.61, 0.76) < 0.0001
TG	0.70 (0.61, 0.80) < 0.0001	0.65 (0.56, 0.76) < 0.0001	0.69 (0.60, 0.80) < 0.0001	0.65 (0.56, 0.76) < 0.0001
HDL	1.02 (0.76, 1.36) 0.9106	1.08 (0.80, 1.45) 0.6083	1.02 (0.76, 1.36) 0.9184	1.08 (0.80, 1.46) 0.6046
LDL	0.87 (0.76, 0.99) 0.0336	0.82 (0.72, 0.95) 0.0062	0.87 (0.76, 0.99) 0.0364	0.82 (0.71, 0.95) 0.0061
NHHR	0.69 (0.61, 0.78) < 0.0001	0.62 (0.54, 0.71) < 0.0001	0.68 (0.60, 0.78) < 0.0001	0.61 (0.53, 0.71) < 0.0001

Model I no adjusted.

Model II adjusted for age(smooth), weight(smooth), height(smooth) and BMI(smooth).

Model III adjusted for history of past illnesses, history of hypertension, history of CHD, history of diabetes, history of previous surgeries, smoking and alcoholism.

Model IV adjusted for age(smooth), weight(smooth), height(smooth), BMI(smooth), history of past illnesses, history of hypertension, history of CHD, history of diabetes, history of previous surgeries, smoking and alcoholism.

PSM, propensity score matching; UL, uterine leiomyoma; OR, odds ratio; CI, confidence interval; WBC, white blood cell count; PLR, platelet-to-lymphocyte ratio; NLR, neutrophil-to-lymphocyte ratio; MLR, monocyte-to-lymphocyte ratio; SII, systemic immune-inflammation index; A/G, albumin-to-globulin ratio; TC, total cholesterol; TG, triglycerides; HDL, high-density lipoprotein cholesterol; LDL, low-density lipoprotein cholesterol; NHHR, non-HDL cholesterol to HDL cholesterol ratio.

### Nonlinear and threshold effects of serum calcium, inflammatory and lipid indicators on UL

**Figure 2:**
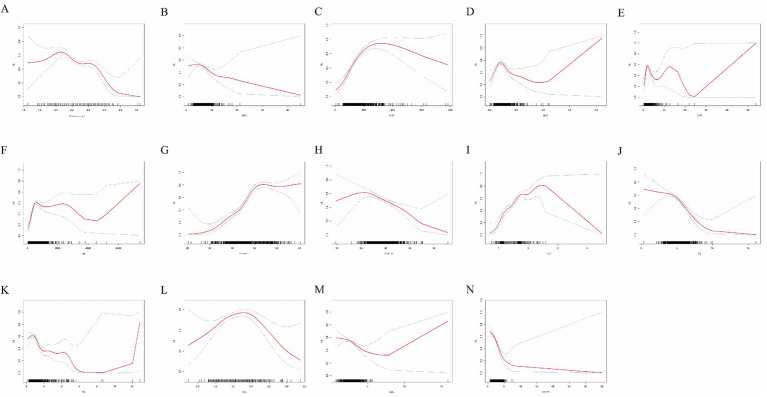
Nonlinear associations between inflammatory, lipid indicators and UL based on smoothing spline models after PSM. **(A)** Serum calcium. **(B)** WBC. **(C)** PLR. **(D)** MLR. **(E)** NLR. **(F)** SII. **(G)** Albumin. **(H)** Globulin. **(I)** A/G. **(J)** TC. **(K)** TG. **(L)** HDL. **(M)** LDL. **(N)** NHHR. The red solid lines represent the estimated effects, and the blue shaded areas indicate the 95% confidence intervals. The distribution of each variable is shown by the rug plot along the x-axis. All analyses were performed after PSM. PSM, propensity score matching; UL, uterine leiomyoma; WBC, white blood cell count; PLR, platelet-to-lymphocyte ratio; NLR, neutrophil-to-lymphocyte ratio; MLR, monocyte-to-lymphocyte ratio; SII, systemic immune-inflammation index; A/G, albumin-to-globulin ratio; TC, total cholesterol; TG, triglycerides; HDL, high-density lipoprotein cholesterol; LDL, low-density lipoprotein cholesterol; NHHR, non-HDL cholesterol to HDL cholesterol ratio.

To further explore the relationship between serum calcium, inflammation, and lipid indicators and the risk of UL occurrence, this study used a smoothing spline curve for nonlinear fitting analysis. The results showed that there was a significant nonlinear correlation between most indicators and UL risk in the population after PSM (Figure 2). Specifically, there is a significant negative correlation between serum calcium and UL risk. As blood calcium levels increase, the risk of UL gradually decreases, and the changes are more pronounced in the lower level range. In the inflammatory indicators, PLR, MLR, SII, etc. show a positive nonlinear relationship with UL risk, with an increase in risk within a certain range as the indicators increase, and a plateau at higher levels. NLR as a whole does not show a significant linear trend. In terms of protein related indicators, the ratio of albumin to A/G is positively correlated with UL risk, while globulin is negatively correlated. In terms of blood lipid indicators, TC, TG, and LDL showed a negative correlation overall, while HDL did not show a significant correlation, and NHHR showed a decreasing trend. The above results also showed a similar trend in the PSM pre analysis (Supplementary Figure 2), indicating consistency in the relationship morphology.

Further analysis of potential threshold effects was conducted using a segmented regression model (Supplementary Tables 6, 7). The results show that most indicators have significant inflection points (K values), indicating that their relationship with UL risk is not simply linear. A threshold effect was identified for serum calcium at approximately 2.48 mmol/L after PSM. Among the 1,402 matched participants, 1,295 had serum calcium levels below 2.48 mmol/L, whereas 107 had levels at or above this threshold. Below the threshold, each 0.1 mmol/L increase in serum calcium was associated with lower odds of UL (OR = 0.88, 95% CI: 0.81–0.96, *p* = 0.0043). Above the threshold, the association remained statistically significant (OR = 0.41, 95% CI: 0.23–0.73, *p* = 0.0027). However, because the number of participants above the threshold was relatively small, the estimated association in this range should be interpreted cautiously. Similarly, PLR, NLR, and MLR exhibit strong risk associations below their respective inflection points, while their effects weaken or tend to stabilize in high-level intervals. In addition, the relationship between TC and TG and UL risk is not significant in the low-level range, but shows a significant protective effect in the high-level range. LDL is positively correlated in the low-level range, but tends to be insignificant in the high-level range. The log likelihood ratio test shows that the segmented models for most indicators outperform the linear models (all *p* < 0.05), further supporting the existence of their nonlinear relationships.

### Mediation analysis of inflammatory and lipid indicators

**Figure 3:**
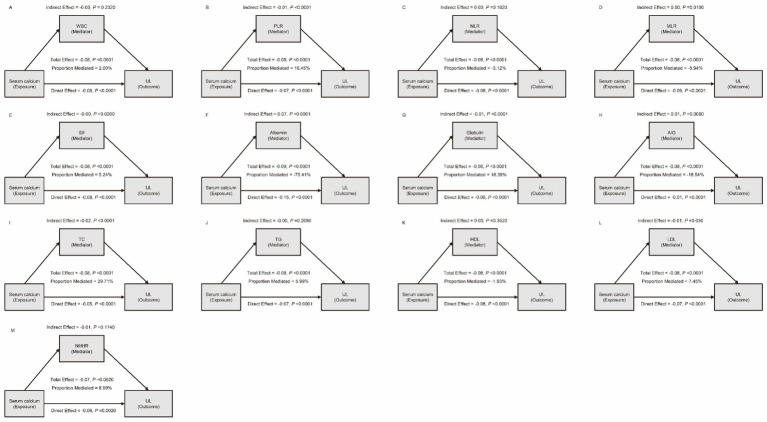
Mediating analysis of the correlation between blood calcium levels and UL using inflammatory and lipid indicators as mediators after PSM. **(A)** WBC. **(B)** PLR. **(C)** NLR. **(D)** MLR. **(E)** SII. **(F)** Albumin. **(G)** Globulin. **(H)** A/G. **(I)** TC. **(J)** TG. **(K)** HDL. **(L)** LDL. **(M)** NHHR. The total effect, direct effect, and indirect (mediation) effect are presented for each outcome. The proportion mediated indicates the percentage of the total effect explained by serum calcium. *p* values were estimated using nonparametric bootstrap methods. Mediation analyses were performed using a probit-based causal mediation model. The reported mediation proportions were derived from the probit scale and should be interpreted with caution, particularly for a binary outcome. Greater emphasis should be placed on the direction and statistical significance of the indirect effects rather than the exact magnitude of the mediation proportions. PSM, propensity score matching; UL, uterine leiomyoma; WBC, white blood cell count; PLR, platelet-to-lymphocyte ratio; NLR, neutrophil-to-lymphocyte ratio; MLR, monocyte-to-lymphocyte ratio; SII, systemic immune-inflammation index; A/G, albumin-to-globulin ratio; TC, total cholesterol; TG, triglycerides; HDL, high-density lipoprotein cholesterol; LDL, low-density lipoprotein cholesterol; NHHR, non-HDL cholesterol to HDL cholesterol ratio.

**Figure 4:**
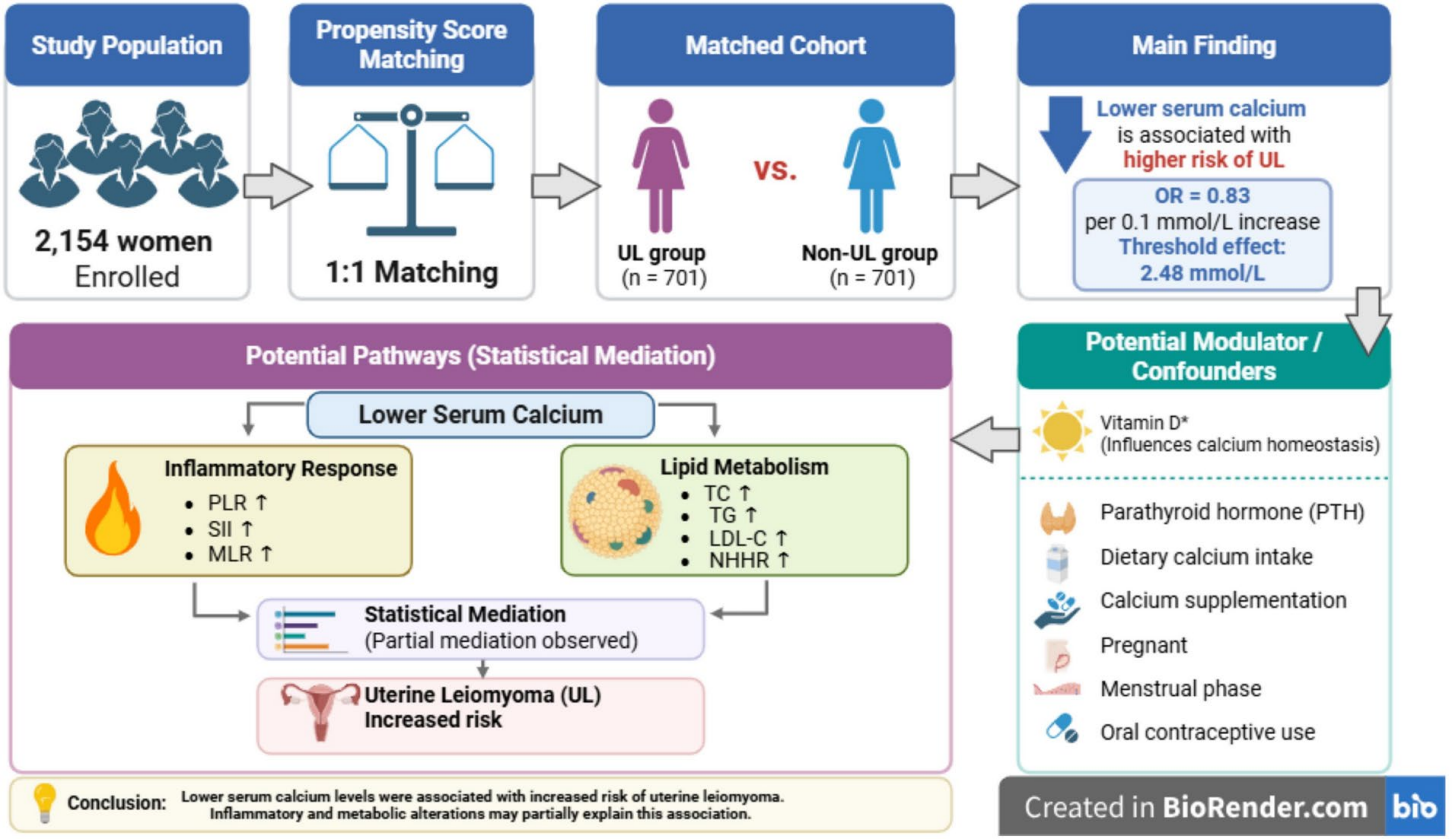
Graphical abstract summarizing the association between serum calcium and uterine leiomyoma. Lower serum calcium levels were associated with increased odds of uterine leiomyoma. Threshold analysis identified an inflection point at approximately 2.48 mmol/L. Inflammatory and lipid-related indicators demonstrated significant statistical mediation effects and may partially explain the observed association. The figure represents a conceptual summary of the study findings rather than confirmed biological mechanisms. UL, uterine leiomyoma; PLR, platelet-to-lymphocyte ratio; MLR, monocyte-to-lymphocyte ratio; SII, systemic immune-inflammation index; TC, total cholesterol; TG, triglycerides; HDL, high-density lipoprotein cholesterol; LDL, low-density lipoprotein cholesterol; NHHR, non-HDL cholesterol to HDL cholesterol ratio. Created in BioRender.com.

To further explore the potential mechanism of the association between serum calcium and UL, this study used inflammation and lipid related indicators as mediator variables to construct a mediation effect model for analysis. The results showed that in the population after PSM, the association between serum calcium and UL showed varying degrees of statistical mediation across multiple inflammatory and lipid indicators. The total effect of serum calcium on UL was statistically significant (*β*: −0.07 to −0.09, all *p* < 0.01), indicating that higher serum calcium levels were associated with lower odds of UL (Figures 3, 4; Table 3). Among the inflammatory indicators, PLR demonstrated a statistically significant indirect effect (indirect effect = −0.01, *p* < 0.001). SII also showed a statistically significant indirect effect (*p* = 0.020). In contrast, the indirect effects of WBC and NLR did not reach statistical significance. Among lipid- and protein-related indicators, TC, Globulin, and LDL also demonstrated significant indirect effects. Albumin and the A/G ratio exhibited indirect effects in the opposite direction to the total effect, suggesting more complex relationships between serum calcium and UL. Overall, the direct effects of most indicators remained statistically significant (all *p* < 0.01), suggesting that the observed association between serum calcium and UL was not fully accounted for by the inflammatory and lipid-related indicators evaluated in this study. Because mediation analyses were performed within a nonlinear modeling framework for a binary outcome, the estimated mediation proportions should be interpreted cautiously. Greater emphasis should be placed on the direction and statistical significance of the indirect effects rather than the exact magnitude of the mediation proportions. Similar patterns were observed in the mediation analyses before PSM (Supplementary Figure 3 and Supplementary Table 8). Although some differences were observed in the estimated mediation proportions, the major indicators demonstrating significant indirect effects and the overall directions of the associations were generally consistent, further supporting the robustness of the findings.

**Table 3:** Intermediary analysis after PSM.

Mediator	Mediation effect, β (95% CI) *p*-value			
	Total effect	Indirect effect	Direct effect	Mediation
WBC	−0.08 (−0.11, −0.05) < 0.0001	−0.00 (−0.01, 0.00) 0.2320	−0.08 (−0.11, −0.05) < 0.0001	2.00%
PLR	−0.08 (−0.12, −0.05) < 0.0001	−0.01 (−0.02, −0.01) < 0.0001	−0.07 (−0.10, −0.04) < 0.0001	16.45%
NLR	−0.08 (−0.12, −0.05) < 0.0001	0.00 (−0.00, 0.01) 0.1620	−0.08 (−0.12, −0.05) < 0.0001	−3.12%
MLR	−0.08 (−0.12, −0.05) < 0.0001	0.00 (0.00, 0.01) 0.0100	−0.09 (−0.12, −0.05) < 0.0001	−5.94%
SII	−0.08 (−0.12, −0.05) < 0.0001	−0.00 (−0.01, −0.00) 0.0200	−0.08 (−0.11, −0.04) < 0.0001	5.24%
Albumin	−0.09 (−0.12, −0.05) < 0.0001	0.07 (0.05, 0.09) < 0.0001	−0.15 (−0.18, −0.12) < 0.0001	−75.41%
Globulin	−0.08 (−0.12, −0.05) < 0.0001	−0.01 (−0.02, −0.01) < 0.0001	−0.06 (−0.10, −0.03) < 0.0001	18.39%
A/G	−0.08 (−0.12, −0.05) < 0.0001	0.01 (0.00, 0.03) 0.0080	−0.10 (−0.13, −0.06) < 0.0001	−18.54%
TC	−0.08 (−0.11, −0.04) < 0.0001	−0.02 (−0.03, −0.01) < 0.0001	−0.05 (−0.09, −0.02) < 0.0001	29.71%
TG	−0.08 (−0.11, −0.04) < 0.0001	−0.00 (−0.01, 0.00) 0.2080	−0.07 (−0.11, −0.04) < 0.0001	5.99%
HDL	−0.08 (−0.11, −0.04) < 0.0001	0.00 (−0.00, 0.01) 0.3520	−0.08 (−0.11, −0.04) < 0.0001	−1.93%
LDL	−0.08 (−0.11, −0.04) < 0.0001	−0.01 (−0.02, −0.00) 0.0300	−0.07 (−0.10, −0.04) < 0.0001	7.45%
NHHR	−0.07 (−0.10, −0.04) 0.0020	−0.01 (−0.02, 0.00) 0.1740	−0.06 (−0.10, −0.03) 0.0020	8.99%

The adjusted variable is age(smooth), weight(smooth), height(smooth), BMI(smooth), history of past illnesses, history of hypertension, history of CHD, history of diabetes, history of previous surgeries, smoking and alcoholism.

Mediation analyses were performed using a probit-based causal mediation model. The reported mediation proportions were derived from the probit scale and should be interpreted with caution, particularly for a binary outcome. Greater emphasis should be placed on the direction and statistical significance of the indirect effects rather than the exact magnitude of the mediation proportions.

PSM, propensity score matching; CI, confidence interval; WBC, white blood cell count; PLR, platelet-to-lymphocyte ratio; NLR, neutrophil-to-lymphocyte ratio; MLR, monocyte-to-lymphocyte ratio; SII, systemic immune-inflammation index; A/G, albumin-to-globulin ratio; TC, total cholesterol; TG, triglycerides; HDL, high-density lipoprotein cholesterol; LDL, low-density lipoprotein cholesterol; NHHR, non-HDL cholesterol to HDL cholesterol ratio.

To assess the potential influence of albumin binding on serum calcium measurements, we additionally performed a sensitivity analysis using albumin-corrected calcium. The inverse association between corrected calcium and UL remained statistically significant before and after PSM across all adjustment models (Supplementary Table 9). These findings suggest that the observed association between serum calcium and UL is unlikely to be explained solely by differences in albumin concentrations.

## Discussion

This study is based on large sample case–control data, systematically evaluating the relationship between serum calcium levels and the risk of UL occurrence, and further exploring the mediating role of inflammation and lipid indicators in it. The results showed a significant negative correlation between serum calcium and UL risk, and this association remained stable after PSM correction, indicating strong independence. In addition, this study found through GAM and segmented regression analysis that there is a significant nonlinear relationship between serum calcium and UL, and a significant threshold effect is observed within a specific level range. Further mediation analysis showed that some inflammation and lipid related indicators played important mediating roles between serum calcium and UL, suggesting that metabolic-inflammatory indicators may statistically explain part of the observed association between serum calcium and UL.

Previous studies on UL have mainly focused on changes in hormone levels, genetic background, and growth factor regulation, with relatively little attention paid to mineral metabolism, especially the role of serum calcium, which still lacks systematic discussion (13, 14). With the increasing attention paid to the relationship between metabolic diseases and gynecological diseases, some studies have observed changes in mineral metabolism status, which may be related to the occurrence of tumor like lesions (14). At the cellular level, calcium ions participate in various signaling processes and affect cell cycle progression and intercellular information transmission. When their homeostasis changes, there may be a shift in the balance between cell proliferation and apoptosis (15, 16). Meanwhile, some studies suggest that there may be a mutual influence between calcium metabolism and inflammatory response. Low calcium status often occurs simultaneously with elevated levels of inflammatory factors, and persistent inflammatory response may also have a feedback effect on calcium homeostasis (17, 18). On another level, the association between changes in lipid metabolism and chronic inflammation has been repeatedly observed in various chronic diseases, but the relevant evidence for the UL population is still limited. Based on the above situation, this study examines serum calcium, inflammatory indicators, and lipid changes within the same analytical framework, in order to understand the overall relationship between these factors and provide reference for further analysis of UL related changes.

Based on existing research and the results of this study, the relationship between serum calcium and UL may involve multiple interrelated biological processes. It should be noted that the present study measured serum total calcium rather than intracellular calcium concentrations or calcium signaling activity. Because serum calcium is tightly regulated within a narrow physiological range, it should be regarded primarily as a systemic biomarker that may reflect broader metabolic, endocrine, or inflammatory states. Therefore, the biological mechanisms discussed below are speculative and should not be interpreted as direct evidence linking serum calcium levels to intracellular calcium signaling pathways. Calcium ions, as an important signal regulator in cells, can affect the signal transmission process in smooth muscle cells and further impact cell contraction and proliferation behavior. This change may be accompanied by abnormal proliferation of uterine myometrial cells (19, 20). On this basis, when the calcium homeostasis deviates, inflammation related pathways may also be activated, and the sustained involvement of the NF - *κ* B pathway is often accompanied by an increase in the release of inflammatory mediators, thus forming a sustained low-grade inflammatory state, which may be consistent with tumor like growth conditions (21–23). At the same time, changes in calcium levels may also affect lipid metabolism related processes, such as by altering enzyme activity or lipoprotein composition, leading to fluctuations in lipid status. In some cases, these changes may occur synchronously with inflammatory responses, forming a process of mutual influence (24, 25). Rather than acting as completely independent processes, calcium homeostasis, lipid metabolism, and inflammatory signaling may interact as components of a broader metabolic-inflammatory network. Altered calcium status may be accompanied by changes in immune cell activity and inflammatory mediator production, whereas chronic inflammation can further influence calcium regulation and lipid metabolism. Similarly, dyslipidemia may contribute to a pro-inflammatory microenvironment that promotes cellular proliferation and extracellular matrix remodeling. Therefore, the observed associations between serum calcium, inflammatory indicators, lipid-related markers, and UL may reflect interconnected biological processes rather than isolated pathways. Nevertheless, the present study was observational in nature, and mechanistic studies are required to validate these hypotheses. Based on the results of this study, GAM showed that the relationship between serum calcium and UL risk did not show a linear change, but rather exhibited more significant fluctuations within specific intervals. This suggests that different levels of serum calcium may correspond to different physiological states or modes of action. Several inflammatory and lipid-related indicators demonstrated significant statistical mediation effects in the association between serum calcium and UL. These findings suggest that inflammatory and metabolic alterations may partially explain the observed relationship between serum calcium levels and UL risk. However, because serum calcium, mediator variables, and UL status were measured contemporaneously, the temporal sequence underlying these associations cannot be determined. Therefore, the observed mediation effects should be interpreted as statistical associations rather than evidence of causal pathways. Although these associations were statistically significant, the absolute difference in serum calcium levels between the UL and control groups was relatively small (approximately 0.04 mmol/L). Therefore, the clinical significance of this difference should be interpreted cautiously, and the present findings alone do not support the use of serum calcium as a standalone clinical screening or diagnostic marker. Future studies are needed to determine whether serum calcium provides meaningful incremental value when incorporated into multivariable risk prediction models. Although the observed associations do not support immediate clinical decision-making, serum calcium, inflammatory indicators, and lipid-related markers are routinely available laboratory measurements and may provide additional information for risk stratification in future studies. The identified threshold effect of serum calcium may also help generate hypotheses regarding potential biological ranges associated with UL risk. However, prospective studies are required to determine whether these biomarkers have predictive value or can be incorporated into clinical screening or prevention strategies. Furthermore, the present findings provide a rationale for future prospective studies evaluating whether calcium metabolism may represent a modifiable pathway associated with UL risk. Nevertheless, the current evidence is insufficient to support routine serum calcium screening or calcium supplementation as preventive strategies in clinical practice.

There are still several limitations to this study. Firstly, this study is a retrospective case–control design. Although PSM was used to reduce confounding bias, residual confounding cannot be completely excluded. Several potentially important variables that may influence both calcium metabolism and UL risk were unavailable in our database, including vitamin D status, parathyroid hormone (PTH), dietary calcium intake, calcium supplement use, menstrual phase, oral contraceptive use, and parity. In particular, vitamin D deficiency has been associated with both altered calcium homeostasis and an increased risk of uterine leiomyoma. Therefore, vitamin D status may represent an important unmeasured confounder and could partially contribute to the observed association between serum calcium and UL. In addition, although we adjusted for a history of past illnesses, this binary variable may not adequately capture the heterogeneous effects of specific chronic diseases on calcium homeostasis and UL risk. Therefore, the observed associations should be interpreted with caution. Furthermore, all exposure, mediator, and outcome variables were measured at the same time point. Therefore, the temporal ordering required for causal mediation analysis cannot be established. The observed mediation effects should be interpreted as statistical mediation rather than causal mediation. Reverse causality remains possible, whereby UL itself may influence inflammatory status, lipid metabolism, and serum calcium levels. Prospective longitudinal studies and mechanistic investigations are needed to clarify the directionality of these associations. Because the study was based on retrospective data, the sample size was determined by available eligible participants rather than by *a priori* power calculation. In particular, no formal power calculation was performed for the mediation analyses. Therefore, the statistical power to detect small indirect effects may be limited. Future prospective studies should incorporate sample size estimation based on predefined effect sizes, especially when mediation effects are of primary interest. Secondly, the detailed clinical characteristics of uterine leiomyoma, such as size, quantity, and classification, are not fully recorded in some cases, which limits further stratified analysis. Prospective studies are needed in the future to verify the stability of associations under different clinical phenotypes. In addition, although some variables have significant statistical differences, their absolute differences are small and may be within the standard error range of the detection method, indicating that their clinical significance still needs to be interpreted with caution. The inflammatory indicators in this study mainly come from routine hematological parameters and their derived indicators, which may not fully reflect the inflammatory status in the local tissue microenvironment. Patients with adenomyosis, endometriosis, and other common benign gynecological diseases were excluded during study design to reduce disease-related heterogeneity. However, these conditions frequently coexist with UL and may share inflammatory and hormonal mechanisms. Therefore, the study population may not fully represent the broader spectrum of patients with UL encountered in routine clinical practice, which may limit the generalizability of the present findings. Future studies including women with coexisting gynecological conditions are needed to validate the robustness and external applicability of these associations. Although threshold analysis suggested an inflection point for serum calcium, only a limited number of participants had serum calcium levels above this threshold. Therefore, the estimated association in the upper serum calcium range may be less stable and should be validated in larger independent populations. Finally, although mediation analysis suggests that inflammation and lipid indicators play a certain role between serum calcium and UL, this study still lacks direct mechanistic evidence, and the relevant molecular pathways need to be further validated through basic experiments and multi omics research.

Lower serum calcium levels were significantly associated with an increased risk of UL, and several inflammatory and lipid-related indicators demonstrated significant statistical mediation effects in this association. These findings suggest that inflammatory and metabolic alterations may partially explain the observed relationship between serum calcium and UL. However, causal interpretations should be avoided because the temporal sequence of these variables cannot be established in the present study. Future prospective and mechanistic studies are warranted to further elucidate the biological pathways linking serum calcium, inflammatory responses, lipid metabolism, and UL.

## Data Availability

The datasets presented in this study can be found in online repositories. The names of the repository/repositories and accession number(s) can be found in the article/Supplementary material.

## Footnotes

1
http://www.R-project.org

## Glossary

UL: uterine leiomyoma
PSM: propensity score matching
GAM: generalized additive model
BMI: body mass index
HB: hemoglobin
PLT: platelet count
LBC: lymphocyte count
NEU: neutrophil count
HDL: high-density lipoprotein.
LDL: low-density lipoprotein.
TC: total cholesterol
TG: triglycerides
PLR: platelet-to-lymphocyte ratio
NLR: neutrophil-to-lymphocyte ratio
MLR: monocyte-to-lymphocyte ratio
SII: systemic immune-inflammation index
A/G: albumin-to-globulin ratio
NHHR: non-high-density lipoprotein to high-density lipoprotein ratio
SD: standard deviation
SMD: standardized mean difference
OR: odds ratio
CI: confidence interval
CHD: coronary heart disease
