# Supplementary material for: Association between serum calcium levels and uterine leiomyoma: a retrospective case–control study exploring the potential mediating roles of inflammatory and lipid-related indicators
Source: Front Nutr. 2026 Jul 22;13:1871649. doi: 10.3389/fnut.2026.1871649 (PMC13437432; doi:10.3389/fnut.2026.1871649)
Supplement: Supplementary file 1 [file Data_sheet_1.docx]

Supplementary table 1. Baseline feature description of the study population before PSM

|  | Total(n=2,154)  Mean±SD / N(%) | No-UL(n=985)  Mean±SD / N(%) | UL(n=1,169)  Mean±SD / N(%) | P value |
| --- | --- | --- | --- | --- |
| Age, years | 44.28 ± 8.92 | 44.30 ± 11.26 | 44.27 ± 6.31 | 0.933 |
| Weight, kg | 64.48 ± 10.18 | 64.59 ± 10.70 | 64.38 ± 9.72 | 0.637 |
| Height, cm | 161.51 ± 5.10 | 162.13 ± 4.87 | 160.99 ± 5.23 | <0.001 |
| BMI, kg/m^2^ | 24.72 ± 3.82 | 24.57 ± 3.90 | 24.85 ± 3.74 | 0.086 |
| History of past illnesses, n (%) |  |  |  | <0.001 |
| No | 1335 (61.98%) | 737 (74.82%) | 598 (51.15%) |  |
| Yes | 819 (38.02%) | 248 (25.18%) | 571 (48.85%) |  |
| History of hypertension, n (%) |  |  |  | <0.001 |
| No | 1893 (87.88%) | 793 (80.51%) | 1100 (94.10%) |  |
| Yes | 261 (12.12%) | 192 (19.49%) | 69 (5.90%) |  |
| History of CHD, n (%) |  |  |  | <0.001 |
| No | 2040 (94.71%) | 876 (88.93%) | 1164 (99.57%) |  |
| Yes | 114 (5.29%) | 109 (11.07%) | 5 (0.43%) |  |
| History of diabetes, n (%) |  |  |  | <0.001 |
| No | 2010 (93.31%) | 867 (88.02%) | 1143 (97.78%) |  |
| Yes | 144 (6.69%) | 118 (11.98%) | 26 (2.22%) |  |
| History of previous surgeries, n (%) |  |  |  | 0.695 |
| No | 1252 (58.12%) | 577 (58.58%) | 675 (57.74%) |  |
| Yes | 902 (41.88%) | 408 (41.42%) | 494 (42.26%) |  |
| Smoking, n (%) |  |  |  | 0.006 |
| No | 2136 (99.16%) | 971 (98.58%) | 1165 (99.66%) |  |
| Yes | 18 (0.84%) | 14 (1.42%) | 4 (0.34%) |  |
| Alcoholism, n (%) |  |  |  | <0.001 |
| No | 2135 (99.12%) | 969 (98.38%) | 1166 (99.74%) |  |
| Yes | 19 (0.88%) | 16 (1.62%) | 3 (0.26%) |  |
| Serum calcium, mmol/L | 2.29 ± 0.14 | 2.31 ± 0.15 | 2.27 ± 0.13 | <0.001 |
| WBC, 10^9^/L | 6.06 ± 2.16 | 6.28 ± 2.51 | 5.88 ± 1.80 | <0.001 |
| RBC, 10^12^/L | 4.37 ± 0.45 | 4.32 ± 0.41 | 4.40 ± 0.47 | <0.001 |
| LBC, 10^9^/L | 1.87 ± 1.00 | 1.94 ± 1.35 | 1.81 ± 0.54 | 0.002 |
| MBC, 10^9^/L | 0.38 ± 0.16 | 0.40 ± 0.18 | 0.37 ± 0.14 | <0.001 |
| NEU, 10^9^/L | 3.69 ± 1.76 | 3.81 ± 1.93 | 3.59 ± 1.59 | 0.003 |
| HB, g/L | 121.09 ± 22.57 | 126.60 ± 17.89 | 116.44 ± 24.94 | <0.001 |
| PLT, 10^9^/L | 258.24 ± 76.73 | 231.16 ± 65.29 | 281.07 ± 78.23 | <0.001 |
| PLR | 153.40 ± 71.48 | 134.56 ± 64.37 | 169.28 ± 73.32 | <0.001 |
| NLR | 2.25 ± 2.01 | 2.30 ± 1.92 | 2.21 ± 2.08 | 0.331 |
| MLR | 0.22 ± 0.13 | 0.23 ± 0.14 | 0.22 ± 0.11 | 0.017 |
| SII | 574.41 ± 454.86 | 530.59 ± 451.17 | 611.34 ± 454.86 | <0.001 |
| Albumin, g/L | 46.41 ± 7.23 | 43.40 ± 6.40 | 48.85 ± 6.95 | <0.001 |
| Globulin, g/L | 28.88 ± 4.19 | 29.49 ± 4.39 | 28.39 ± 3.97 | <0.001 |
| A/G | 1.52 ± 0.28 | 1.45 ± 0.28 | 1.57 ± 0.26 | <0.001 |
| TC, mmol/L | 5.08 ± 1.13 | 5.32 ± 1.23 | 4.90 ± 1.01 | <0.001 |
| TG, mmol/L | 1.26 ± 1.04 | 1.42 ± 1.11 | 1.13 ± 0.97 | <0.001 |
| HDL, mmol/L | 1.54 ± 0.37 | 1.53 ± 0.40 | 1.54 ± 0.34 | 0.281 |
| LDL, mmol/L | 2.82 ± 0.85 | 2.90 ± 0.83 | 2.77 ± 0.86 | <0.001 |
| NHHR | 2.45 ± 1.20 | 2.66 ± 1.47 | 2.29 ± 0.91 | <0.001 |

Abbreviations: PSM, propensity score matching; UL, uterine leiomyoma; SD, standard deviation; BMI, body Mass Index; CHD, coronary heart disease; WBC, white blood cell count; RBC, red blood cell count; LBC, lymphocyte count; MBC, monocyte count; NEU, neutrophil count; HB, hemoglobin; PLT, platelet count; PLR, platelet-to-lymphocyte ratio; NLR, neutrophil-to-lymphocyte ratio; MLR, monocyte-to-lymphocyte ratio; SII, systemic immune-inflammation index; A/G, albumin-to-globulin ratio; TC, total cholesterol; TG, triglycerides; HDL, high-density lipoprotein cholesterol; LDL, low-density lipoprotein cholesterol; NHHR, non-HDL cholesterol to HDL cholesterol ratio.

Supplementary table 2. UL single factor analysis before and after PSM

|  | Before PSM  OR (95%CI) P value | After PSM  OR (95%CI) P value |
| --- | --- | --- |
| Age, years | 1.00 (0.99, 1.01) 0.9333 | 1.01 (1.00, 1.02) 0.0414 |
| Weight, kg | 1.00 (0.99, 1.01) 0.6367 | 1.00 (0.99, 1.01) 0.6506 |
| Height, cm | 0.96 (0.94, 0.97) <0.0001 | 0.97 (0.95, 0.99) 0.0121 |
| BMI, kg/m^2^ | 1.02 (1.00, 1.04) 0.0862 | 1.01 (0.98, 1.04) 0.4745 |
| History of past illnesses, n (%) |  |  |
| No | 1.0 | 1.0 |
| Yes | 2.84 (2.36, 3.41) <0.0001 | 1.31 (1.04, 1.66) 0.0215 |
| History of hypertension, n (%) |  |  |
| No | 1.0 | 1.0 |
| Yes | 0.26 (0.19, 0.35) <0.0001 | 1.19 (0.79, 1.79) 0.4069 |
| History of CHD, n (%) |  |  |
| No | 1.0 | 1.0 |
| Yes | 0.03 (0.01, 0.08) <0.0001 | 1.00 (0.29, 3.47) 1.0000 |
| History of diabetes, n (%) |  |  |
| No | 1.0 | 1.0 |
| Yes | 0.17 (0.11, 0.26) <0.0001 | 0.96 (0.54, 1.70) 0.8844 |
| History of previous surgeries, n (%) |  |  |
| No | 1.0 | 1.0 |
| Yes | 1.03 (0.87, 1.23) 0.6949 | 0.81 (0.64, 1.02) 0.0749 |
| Smoking, n (%) |  |  |
| No | 1.0 | 1.0 |
| Yes | 0.24 (0.08, 0.73) 0.0116 | 1.00 (0.25, 4.01) 1.0000 |
| Alcoholism, n (%) |  |  |
| No | 1.0 | 1.0 |
| Yes | 0.16 (0.05, 0.54) 0.0032 | 0.75 (0.17, 3.36) 0.7057 |
| Serum calcium, mmol/L  (per 0.1 mmol/L increase) | 0.82 (0.77, 0.87) <0.0001 | 0.83 (0.77, 0.90) <0.0001 |
| WBC, 10^9^/L | 0.91 (0.87, 0.95) <0.0001 | 0.92 (0.87, 0.97) 0.0013 |
| RBC, 10^12^/L | 1.49 (1.23, 1.80) <0.0001 | 1.46 (1.15, 1.86) 0.0020 |
| LBC, 10^9^/L | 0.76 (0.66, 0.87) 0.0002 | 0.81 (0.68, 0.97) 0.0200 |
| MBC, 10^9^/L | 0.30 (0.17, 0.51) <0.0001 | 0.30 (0.15, 0.59) 0.0006 |
| NEU, 10^9^/L | 0.93 (0.89, 0.98) 0.0036 | 0.93 (0.88, 0.99) 0.0171 |
| HB, g/L | 0.98 (0.97, 0.98) <0.0001 | 0.98 (0.97, 0.98) <0.0001 |
| PLT, 10^9^/L | 1.01 (1.01, 1.01) <0.0001 | 1.01 (1.01, 1.01) <0.0001 |
| PLR | 1.01 (1.01, 1.01) <0.0001 | 1.01 (1.01, 1.01) <0.0001 |
| NLR | 0.98 (0.94, 1.02) 0.3380 | 0.98 (0.93, 1.03) 0.3478 |
| MLR | 0.44 (0.22, 0.87) 0.0189 | 0.42 (0.18, 0.98) 0.0442 |
| SII | 1.00 (1.00, 1.00) <0.0001 | 1.00 (1.00, 1.00) 0.0092 |
| Albumin, g/L | 1.13 (1.11, 1.15) <0.0001 | 1.14 (1.12, 1.16) <0.0001 |
| Globulin, g/L | 0.94 (0.92, 0.96) <0.0001 | 0.94 (0.91, 0.96) <0.0001 |
| A/G | 6.04 (4.24, 8.60) <0.0001 | 6.00 (3.91, 9.21) <0.0001 |
| TC, mmol/L | 0.70 (0.65, 0.77) <0.0001 | 0.71 (0.64, 0.79) <0.0001 |
| TG, mmol/L | 0.72 (0.65, 0.80) <0.0001 | 0.70 (0.61, 0.80) <0.0001 |
| HDL, mmol/L | 1.14 (0.90, 1.45) 0.2812 | 1.02 (0.76, 1.36) 0.9106 |
| LDL, mmol/L | 0.83 (0.75, 0.93) 0.0009 | 0.87 (0.76, 0.99) 0.0336 |
| NHHR | 0.66 (0.60, 0.73) <0.0001 | 0.69 (0.61, 0.78) <0.0001 |

Abbreviations: PSM, propensity score matching; UL, uterine leiomyoma; OR, odds ratio; CI, confidence interval; BMI, body Mass Index; CHD, coronary heart disease; WBC, white blood cell count; RBC, red blood cell count; LBC, lymphocyte count; MBC, monocyte count; NEU, neutrophil count; HB, hemoglobin; PLT, platelet count; PLR, platelet-to-lymphocyte ratio; NLR, neutrophil-to-lymphocyte ratio; MLR, monocyte-to-lymphocyte ratio; SII, systemic immune-inflammation index; A/G, albumin-to-globulin ratio; TC, total cholesterol; TG, triglycerides; HDL, high-density lipoprotein cholesterol; LDL, low-density lipoprotein cholesterol; NHHR, non-HDL cholesterol to HDL cholesterol ratio.

Supplementary table 3. UL multiple regression analysis before PSM

| Exposure | Model I  OR (95%CI) Pvalue | Model II  OR (95%CI) Pvalue | Model III  OR (95%CI) Pvalue | Model IV  OR (95%CI) Pvalue |
| --- | --- | --- | --- | --- |
| Serum calcium  (per 0.1 mmol/L increase) | 0.82 (0.77, 0.87) <0.0001 | 0.82 (0.77, 0.88) <0.0001 | 0.85 (0.79, 0.91) <0.0001 | 0.85 (0.79, 0.91) <0.0001 |
| WBC | 0.91 (0.87, 0.95) <0.0001 | 0.90 (0.86, 0.95) <0.0001 | 0.93 (0.88, 0.97) 0.0015 | 0.93 (0.89, 0.98) 0.0035 |
| PLR | 1.01 (1.01, 1.01) <0.0001 | 1.01 (1.01, 1.01) <0.0001 | 1.01 (1.01, 1.01) <0.0001 | 1.01 (1.01, 1.01) <0.0001 |
| NLR | 0.98 (0.94, 1.02) 0.3380 | 0.98 (0.93, 1.02) 0.3060 | 0.97 (0.93, 1.02) 0.2908 | 0.98 (0.94, 1.03) 0.4003 |
| MLR | 0.44 (0.22, 0.87) 0.0189 | 0.45 (0.22, 0.89) 0.0229 | 0.35 (0.16, 0.76) 0.0085 | 0.40 (0.18, 0.87) 0.0213 |
| SII | 1.00 (1.00, 1.00) <0.0001 | 1.00 (1.00, 1.00) 0.0001 | 1.00 (1.00, 1.00) 0.0012 | 1.00 (1.00, 1.00) 0.0006 |
| Albumin | 1.13 (1.11, 1.15) <0.0001 | 1.14 (1.12, 1.15) <0.0001 | 1.14 (1.12, 1.16) <0.0001 | 1.14 (1.12, 1.16) <0.0001 |
| Globulin | 0.94 (0.92, 0.96) <0.0001 | 0.94 (0.92, 0.96) <0.0001 | 0.94 (0.92, 0.97) <0.0001 | 0.94 (0.92, 0.96) <0.0001 |
| A/G | 6.04 (4.24, 8.60) <0.0001 | 6.48 (4.52, 9.28) <0.0001 | 5.96 (4.05, 8.79) <0.0001 | 6.64 (4.46, 9.88) <0.0001 |
| TC | 0.70 (0.65, 0.77) <0.0001 | 0.69 (0.63, 0.75) <0.0001 | 0.74 (0.68, 0.81) <0.0001 | 0.70 (0.64, 0.77) <0.0001 |
| TG | 0.72 (0.65, 0.80) <0.0001 | 0.67 (0.60, 0.76) <0.0001 | 0.79 (0.70, 0.88) <0.0001 | 0.73 (0.65, 0.83) <0.0001 |
| HDL | 1.14 (0.90, 1.45) 0.2812 | 1.19 (0.93, 1.53) 0.1647 | 0.99 (0.76, 1.29) 0.9228 | 1.11 (0.84, 1.46) 0.4689 |
| LDL | 0.83 (0.75, 0.93) 0.0009 | 0.82 (0.73, 0.92) 0.0006 | 0.90 (0.80, 1.01) 0.0837 | 0.84 (0.74, 0.95) 0.0065 |
| NHHR | 0.66 (0.60, 0.73) <0.0001 | 0.61 (0.54, 0.68) <0.0001 | 0.74 (0.66, 0.83) <0.0001 | 0.65 (0.58, 0.74) <0.0001 |

Model I no adjusted.

Model II adjusted for age(smooth), weight(smooth), height(smooth) and BMI(smooth).

Model III adjusted for history of past illnesses, history of hypertension, history of CHD, history of diabetes, history of previous surgeries, smoking and alcoholism.

Model IV adjusted for age(smooth), weight(smooth), height(smooth), BMI(smooth), history of past illnesses, history of hypertension, history of CHD, history of diabetes, history of previous surgeries, smoking and alcoholism.

Abbreviations: PSM, propensity score matching; UL, uterine leiomyoma; OR, odds ratio; CI, confidence interval; WBC, white blood cell count; PLR, platelet-to-lymphocyte ratio; NLR, neutrophil-to-lymphocyte ratio; MLR, monocyte-to-lymphocyte ratio; SII, systemic immune-inflammation index; A/G, albumin-to-globulin ratio; TC, total cholesterol; TG, triglycerides; HDL, high-density lipoprotein cholesterol; LDL, low-density lipoprotein cholesterol; NHHR, non-HDL cholesterol to HDL cholesterol ratio.

Supplementary table 4. Multivariate regression analysis of serum calcium, inflammation and lipid indicators before PSM

| Exposure | Model I  OR (95%CI) Pvalue | Model II  OR (95%CI) Pvalue | Model III  OR (95%CI) Pvalue | Model IV  OR (95%CI) Pvalue |
| --- | --- | --- | --- | --- |
| WBC | 0.43 (-0.21, 1.08) 0.1888 | 0.80 (0.16, 1.44) 0.0142 | 0.33 (-0.32, 0.98) 0.3165 | 0.67 (0.03, 1.31) 0.0409 |
| PLR | -44.97 (-66.18, -23.75) <0.0001 | -45.92 (-67.32, -24.53) <0.0001 | -36.65 (-57.85, -15.44) 0.0007 | -38.59 (-59.94, -17.23) 0.0004 |
| NLR | -1.21 (-1.81, -0.62) <0.0001 | -1.08 (-1.68, -0.48) 0.0004 | -1.19 (-1.80, -0.59) 0.0001 | -1.10 (-1.70, -0.49) 0.0004 |
| MLR | -0.09 (-0.13, -0.06) <0.0001 | -0.09 (-0.13, -0.05) <0.0001 | -0.09 (-0.13, -0.05) <0.0001 | -0.09 (-0.13, -0.05) <0.0001 |
| SII | -250.72 (-385.84, -115.60) 0.0003 | -213.70 (-349.65, -77.75) 0.0021 | -227.20 (-363.53, -90.87) 0.0011 | -199.37 (-336.33, -62.42) 0.0044 |
| Albumin | 11.72 (9.58, 13.85) <0.0001 | 11.33 (9.19, 13.48) <0.0001 | 12.20 (10.08, 14.32) <0.0001 | 11.82 (9.68, 13.95) <0.0001 |
| Globulin | 6.63 (5.40, 7.87) <0.0001 | 6.83 (5.59, 8.07) <0.0001 | 6.50 (5.25, 7.74) <0.0001 | 6.74 (5.49, 7.99) <0.0001 |
| A/G | 0.14 (0.06, 0.23) 0.0008 | 0.13 (0.05, 0.21) 0.0025 | 0.16 (0.08, 0.24) 0.0002 | 0.14 (0.06, 0.22) 0.0011 |
| TC | 1.83 (1.49, 2.17) <0.0001 | 1.74 (1.41, 2.08) <0.0001 | 1.74 (1.40, 2.08) <0.0001 | 1.71 (1.37, 2.05) <0.0001 |
| TG | 0.37 (0.05, 0.69) 0.0228 | 0.47 (0.16, 0.79) 0.0030 | 0.23 (-0.09, 0.55) 0.1635 | 0.38 (0.06, 0.69) 0.0187 |
| HDL | 0.27 (0.16, 0.38) <0.0001 | 0.27 (0.16, 0.38) <0.0001 | 0.32 (0.20, 0.43) <0.0001 | 0.29 (0.18, 0.40) <0.0001 |
| LDL | 1.20 (0.94, 1.46) <0.0001 | 1.11 (0.86, 1.36) <0.0001 | 1.15 (0.90, 1.41) <0.0001 | 1.12 (0.87, 1.37) <0.0001 |
| NHHR | 0.53 (0.16, 0.90) 0.0049 | 0.48 (0.12, 0.84) 0.0086 | 0.37 (-0.00, 0.73) 0.0523 | 0.40 (0.04, 0.76) 0.0304 |

Model I no adjusted.

Model II adjusted for age(smooth), weight(smooth), height(smooth) and BMI(smooth).

Model III adjusted for history of past illnesses, history of hypertension, history of CHD, history of diabetes, history of previous surgeries, smoking and alcoholism.

Model IV adjusted for age(smooth), weight(smooth), height(smooth), BMI(smooth), history of past illnesses, history of hypertension, history of CHD, history of diabetes, history of previous surgeries, smoking and alcoholism.

Abbreviations: PSM, propensity score matching; OR, odds ratio; CI, confidence interval; WBC, white blood cell count; PLR, platelet-to-lymphocyte ratio; NLR, neutrophil-to-lymphocyte ratio; MLR, monocyte-to-lymphocyte ratio; SII, systemic immune-inflammation index; A/G, albumin-to-globulin ratio; TC, total cholesterol; TG, triglycerides; HDL, high-density lipoprotein cholesterol; LDL, low-density lipoprotein cholesterol; NHHR, non-HDL cholesterol to HDL cholesterol ratio.

Supplementary table 5. Multivariate regression analysis of serum calcium, inflammation and lipid indicators after PSM

| Exposure | Model I  OR (95%CI) Pvalue | Model II  OR (95%CI) Pvalue | Model III  OR (95%CI) Pvalue | Model IV  OR (95%CI) Pvalue |
| --- | --- | --- | --- | --- |
| WBC | 0.11 (-0.71, 0.93) 0.7947 | 0.53 (-0.29, 1.34) 0.2032 | 0.08 (-0.75, 0.91) 0.8552 | 0.47 (-0.35, 1.29) 0.2579 |
| PLR | -51.04 (-77.77, -24.30) 0.0002 | -54.70 (-81.63, -27.77) <0.0001 | -48.25 (-75.15, -21.35) 0.0005 | -52.08 (-79.18, -24.99) 0.0002 |
| NLR | -1.82 (-2.60, -1.05) <0.0001 | -1.64 (-2.42, -0.86) <0.0001 | -1.83 (-2.61, -1.04) <0.0001 | -1.66 (-2.45, -0.88) <0.0001 |
| MLR | -0.11 (-0.15, -0.06) <0.0001 | -0.10 (-0.15, -0.06) <0.0001 | -0.10 (-0.15, -0.05) <0.0001 | -0.10 (-0.15, -0.05) <0.0001 |
| SII | -367.52 (-542.05, -192.99) <0.0001 | -328.67 (-504.17, -153.18) 0.0003 | -362.62 (-538.25, -187.00) <0.0001 | -327.90 (-504.41, -151.38) 0.0003 |
| Albumin | 12.30 (9.69, 14.91) <0.0001 | 11.62 (9.00, 14.25) <0.0001 | 12.53 (9.91, 15.15) <0.0001 | 11.88 (9.24, 14.52) <0.0001 |
| Globulin | 5.93 (4.40, 7.45) <0.0001 | 6.10 (4.57, 7.63) <0.0001 | 5.75 (4.21, 7.28) <0.0001 | 5.95 (4.41, 7.49) <0.0001 |
| A/G | 0.20 (0.10, 0.30) 0.0002 | 0.18 (0.08, 0.29) 0.0006 | 0.21 (0.11, 0.32) <0.0001 | 0.19 (0.09, 0.30) 0.0003 |
| TC | 1.59 (1.17, 2.00) <0.0001 | 1.52 (1.11, 1.93) <0.0001 | 1.54 (1.12, 1.96) <0.0001 | 1.50 (1.08, 1.91) <0.0001 |
| TG | 0.20 (-0.16, 0.57) 0.2673 | 0.35 (-0.00, 0.70) 0.0528 | 0.13 (-0.23, 0.49) 0.4721 | 0.30 (-0.05, 0.65) 0.0967 |
| HDL | 0.23 (0.09, 0.37) 0.0015 | 0.23 (0.09, 0.36) 0.0012 | 0.25 (0.11, 0.39) 0.0005 | 0.24 (0.10, 0.38) 0.0008 |
| LDL | 1.04 (0.71, 1.36) <0.0001 | 0.94 (0.63, 1.26) <0.0001 | 1.01 (0.68, 1.33) <0.0001 | 0.93 (0.62, 1.25) <0.0001 |
| NHHR | 0.44 (-0.04, 0.92) 0.0731 | 0.38 (-0.08, 0.85) 0.1080 | 0.36 (-0.12, 0.84) 0.1452 | 0.34 (-0.13, 0.81) 0.1558 |

Model I no adjusted.

Model II adjusted for age(smooth), weight(smooth), height(smooth) and BMI(smooth).

Model III adjusted for history of past illnesses, history of hypertension, history of CHD, history of diabetes, history of previous surgeries, smoking and alcoholism.

Model IV adjusted for age(smooth), weight(smooth), height(smooth), BMI(smooth), history of past illnesses, history of hypertension, history of CHD, history of diabetes, history of previous surgeries, smoking and alcoholism.

Abbreviations: PSM, propensity score matching; OR, odds ratio; CI, confidence interval; WBC, white blood cell count; PLR, platelet-to-lymphocyte ratio; NLR, neutrophil-to-lymphocyte ratio; MLR, monocyte-to-lymphocyte ratio; SII, systemic immune-inflammation index; A/G, albumin-to-globulin ratio; TC, total cholesterol; TG, triglycerides; HDL, high-density lipoprotein cholesterol; LDL, low-density lipoprotein cholesterol; NHHR, non-HDL cholesterol to HDL cholesterol ratio.

Supplementary table 6. Saturation threshold effect analysis before PSM

| Exposure: | Serum calcium  (per 0.1 mmol/L increase) | WBC | PLR | NLR | MLR | SII | Albumin | Globulin | A/G | TC | TG | HDL | LDL | NHHR |
| --- | --- | --- | --- | --- | --- | --- | --- | --- | --- | --- | --- | --- | --- | --- |
| Model I |  |  |  |  |  |  |  |  |  |  |  |  |  |  |
| A straight line effect | 0.85 (0.79, 0.91) <0.0001 | 0.93 (0.89, 0.98) 0.0035 | 1.01 (1.01, 1.01) <0.0001 | 0.98 (0.94, 1.03) 0.4003 | 0.40 (0.18, 0.87) 0.0213 | 1.00 (1.00, 1.00) 0.0006 | 1.14 (1.12, 1.16) <0.0001 | 0.94 (0.92, 0.96) <0.0001 | 6.64 (4.46, 9.88) <0.0001 | 0.70 (0.64, 0.77) <0.0001 | 0.73 (0.65, 0.83) <0.0001 | 1.11 (0.84, 1.46) 0.4689 | 0.84 (0.74, 0.95) 0.0065 | 0.65 (0.58, 0.74) <0.0001 |
| Model II |  |  |  |  |  |  |  |  |  |  |  |  |  |  |
| Folding point (K) | 2.50 | 3.98 | 214.95 | 1.36 | 0.17 | 392.62 | 52.4 | 22.34 | 1.8 | 4.58 | 1.67 | 1.85 | 4.19 | 4.05 |
| <K-segment effect 1 | 0.90 (0.84, 0.98) 0.0099 | 1.84 (1.17, 2.91) 0.0088 | 1.01 (1.01, 1.02) <0.0001 | 3.97 (2.10, 7.51) <0.0001 | 1368.79 (29.27, 64003.58) 0.0002 | 1.01 (1.01, 1.01) <0.0001 | 1.19 (1.16, 1.22) <0.0001 | 1.17 (0.97, 1.41) 0.0924 | 11.76 (7.14, 19.38) <0.0001 | 0.98 (0.75, 1.29) 0.8972 | 0.43 (0.33, 0.56) <0.0001 | 2.10 (1.42, 3.11) 0.0002 | 0.76 (0.66, 0.89) 0.0004 | 0.59 (0.51, 0.68) <0.0001 |
| >K-segment effect 2 | 0.30 (0.17, 0.56) 0.0001 | 0.90 (0.86, 0.95) 0.0002 | 1.00 (1.00, 1.00) 0.1789 | 0.95 (0.89, 1.00) 0.0530 | 0.14 (0.05, 0.37) <0.0001 | 1.00 (1.00, 1.00) 0.4628 | 0.99 (0.94, 1.05) 0.8193 | 0.93 (0.91, 0.96) <0.0001 | 0.77 (0.28, 2.11) 0.6067 | 0.62 (0.54, 0.71) <0.0001 | 0.93 (0.82, 1.06) 0.2930 | 0.23 (0.11, 0.49) 0.0001 | 1.17 (0.83, 1.65) 0.3730 | 0.94 (0.80, 1.10) 0.4551 |
| The difference in effect between 2 and 1 | 0.34 (0.18, 0.63) 0.0007 | 0.49 (0.31, 0.79) 0.0032 | 0.98 (0.98, 0.99) <0.0001 | 0.24 (0.12, 0.46) <0.0001 | 0.00 (0.00, 0.01) <0.0001 | 0.99 (0.99, 0.99) <0.0001 | 0.83 (0.77, 0.90) <0.0001 | 0.79 (0.65, 0.96) 0.0196 | 0.07 (0.02, 0.23) <0.0001 | 0.63 (0.45, 0.89) 0.0091 | 2.17 (1.56, 3.02) <0.0001 | 0.11 (0.04, 0.29) <0.0001 | 1.53 (1.02, 2.29) 0.0380 | 1.59 (1.27, 2.00) <0.0001 |
| Predicted value of equation at inflection point | -0.09 (-0.27, 0.08) | 0.45 (0.31, 0.59) | 1.27 (1.07, 1.47) | 0.33 (0.22, 0.44) | 0.42 (0.30, 0.54) | 0.47 (0.34, 0.59) | 1.36 (1.14, 1.57) | 0.71 (0.53, 0.89) | 0.95 (0.78, 1.12) | 0.58 (0.44, 0.72) | -0.16 (-0.33, 0.01) | 0.57 (0.41, 0.73) | -0.09 (-0.28, 0.11) | -0.51 (-0.72, -0.30) |
| Log Likelihood Ratio Tests | <0.001 | 0.003 | <0.001 | <0.001 | <0.001 | <0.001 | <0.001 | 0.022 | <0.001 | 0.009 | <0.001 | <0.001 | 0.024 | 0.004 |

The adjusted variable is age(smooth), weight(smooth), height(smooth), BMI(smooth), history of past illnesses, history of hypertension, history of CHD, history of diabetes, history of previous surgeries, smoking and alcoholism.

Abbreviations: PSM, propensity score matching; WBC, white blood cell count; PLR, platelet-to-lymphocyte ratio; NLR, neutrophil-to-lymphocyte ratio; MLR, monocyte-to-lymphocyte ratio; SII, systemic immune-inflammation index; A/G, albumin-to-globulin ratio; TC, total cholesterol; TG, triglycerides; HDL, high-density lipoprotein cholesterol; LDL, low-density lipoprotein cholesterol; NHHR, non-HDL cholesterol to HDL cholesterol ratio.

Supplementary table 7. Saturation threshold effect analysis after PSM

| Exposure: | Serum calcium  (per 0.1 mmol/L increase) | WBC | PLR | NLR | MLR | SII | Albumin | Globulin | A/G | TC | TG | HDL | LDL | NHHR |
| --- | --- | --- | --- | --- | --- | --- | --- | --- | --- | --- | --- | --- | --- | --- |
| Model I |  |  |  |  |  |  |  |  |  |  |  |  |  |  |
| A straight line effect | 0.83 (0.77, 0.90) <0.0001 | 0.92 (0.87, 0.97) 0.0036 | 1.01 (1.01, 1.01) <0.0001 | 0.98 (0.93, 1.03) 0.4260 | 0.42 (0.18, 1.00) 0.0508 | 1.00 (1.00, 1.00) 0.0056 | 1.14 (1.12, 1.16) <0.0001 | 0.94 (0.91, 0.96) <0.0001 | 6.64 (4.27, 10.33) <0.0001 | 0.68 (0.61, 0.76) <0.0001 | 0.65 (0.56, 0.76) <0.0001 | 1.08 (0.80, 1.46) 0.6046 | 0.82 (0.71, 0.95) 0.0061 | 0.61 (0.53, 0.71) <0.0001 |
| Model II |  |  |  |  |  |  |  |  |  |  |  |  |  |  |
| Folding point (K) | 2.48 | 4.02 | 217.75 | 1.39 | 0.16 | 428.9 | 52.3 | 22.5 | 1.7 | 4.63 | 1.79 | 1.85 | 4.2 | 1.49 |
| <K-segment effect 1 | 0.88 (0.81, 0.96) 0.0043 | 1.46 (0.93, 2.29) 0.0964 | 1.01 (1.01, 1.02) <0.0001 | 4.62 (2.33, 9.13) <0.0001 | 27180.98 (155.68, 4745798.36) 0.0001 | 1.01 (1.00, 1.01) <0.0001 | 1.20 (1.16, 1.23) <0.0001 | 1.15 (0.94, 1.39) 0.1664 | 15.31 (8.21, 28.53) <0.0001 | 1.06 (0.80, 1.40) 0.6821 | 0.45 (0.34, 0.60) <0.0001 | 2.26 (1.47, 3.50) 0.0002 | 0.74 (0.63, 0.88) 0.0004 | 1.94 (0.76, 4.90) 0.1638 |
| >K-segment effect 2 | 0.41 (0.23, 0.73) 0.0027 | 0.90 (0.85, 0.96) 0.0007 | 1.00 (1.00, 1.00) 0.2271 | 0.93 (0.87, 1.00) 0.0541 | 0.14 (0.05, 0.41) 0.0003 | 1.00 (1.00, 1.00) 0.2304 | 0.98 (0.93, 1.05) 0.6139 | 0.92 (0.90, 0.95) <0.0001 | 1.07 (0.44, 2.59) 0.8866 | 0.56 (0.47, 0.66) <0.0001 | 0.86 (0.70, 1.06) 0.1565 | 0.18 (0.07, 0.43) 0.0001 | 1.15 (0.82, 1.62) 0.4252 | 0.57 (0.48, 0.66) <0.0001 |
| The difference in effect between 2 and 1 | 0.47 (0.25, 0.86) 0.0140 | 0.62 (0.39, 0.98) 0.0424 | 0.98 (0.98, 0.99) <0.0001 | 0.20 (0.10, 0.41) <0.0001 | 0.00 (0.00, 0.00) <0.0001 | 0.99 (0.99, 1.00) <0.0001 | 0.82 (0.76, 0.89) <0.0001 | 0.81 (0.66, 0.99) 0.0380 | 0.07 (0.02, 0.24) <0.0001 | 0.53 (0.36, 0.77) 0.0008 | 1.92 (1.28, 2.88) 0.0017 | 0.08 (0.03, 0.24) <0.0001 | 1.54 (1.03, 2.31) 0.0358 | 0.29 (0.11, 0.78) 0.0146 |
| Predicted value of equation at inflection point | -0.20 (-0.41, 0.00) | 0.26 (0.09, 0.42) | 1.02 (0.78, 1.27) | 0.19 (0.05, 0.33) | 0.24 (0.10, 0.39) | 0.35 (0.20, 0.51) | 1.33 (1.07, 1.60) | 0.54 (0.32, 0.76) | 0.64 (0.45, 0.83) | 0.45 (0.27, 0.63) | -0.32 (-0.54, -0.09) | 0.44 (0.24, 0.64) | -0.21 (-0.45, 0.03) | 0.57 (0.39, 0.76) |
| Log Likelihood Ratio Tests | 0.004 | 0.039 | <0.001 | <0.001 | <0.001 | <0.001 | <0.001 | 0.031 | <0.001 | <0.001 | 0.003 | <0.001 | 0.028 | 0.015 |

The adjusted variable is age(smooth), weight(smooth), height(smooth), BMI(smooth), history of past illnesses, history of hypertension, history of CHD, history of diabetes, history of previous surgeries, smoking and alcoholism.

Abbreviations: PSM, propensity score matching; WBC, white blood cell count; PLR, platelet-to-lymphocyte ratio; NLR, neutrophil-to-lymphocyte ratio; MLR, monocyte-to-lymphocyte ratio; SII, systemic immune-inflammation index; A/G, albumin-to-globulin ratio; TC, total cholesterol; TG, triglycerides; HDL, high-density lipoprotein cholesterol; LDL, low-density lipoprotein cholesterol; NHHR, non-HDL cholesterol to HDL cholesterol ratio.

Supplementary table 8. Intermediary analysis before PSM

| Mediator | Mediation effect, β (95%CI) Pvalue | | | |
| --- | --- | --- | --- | --- |
|  | Total effect | Indirect effect | Direct effect | Mediation |
| WBC | -0.06(-0.08, -0.03) <0.0001 | -0.00(-0.00, 0.00) 0.0620 | -0.06(-0.08, -0.03) <0.0001 | 2.79% |
| PLR | -0.06(-0.08, -0.03) <0.0001 | -0.01(-0.01, -0.00) 0.0020 | -0.05(-0.07, -0.02) <0.0001 | 15.31% |
| NLR | -0.06(-0.08, -0.03) <0.0001 | 0.00(-0.00, 0.00) 0.2120 | -0.06(-0.08, -0.03) <0.0001 | -2.02% |
| MLR | -0.06(-0.08, -0.03) <0.0001 | 0.00(0.00, 0.01) 0.0020 | -0.06(-0.09, -0.04) <0.0001 | -6.26% |
| SII | -0.06(-0.08, -0.03) <0.0001 | -0.00(-0.01, -0.00) 0.0420 | -0.05(-0.08, -0.03) <0.0001 | 4.55% |
| Albumin | -0.07(-0.09, -0.04) <0.0001 | 0.06(0.04, 0.07) <0.0001 | -0.12(-0.14, -0.09) <0.0001 | -84.83% |
| Globulin | -0.06(-0.08, -0.03) <0.0001 | -0.01(-0.02, -0.01) <0.0001 | -0.04(-0.07, -0.02) <0.0001 | 21.19% |
| A/G | -0.06(-0.09, -0.03) <0.0001 | 0.01(0.00, 0.02) 0.0060 | -0.07(-0.09, -0.04) <0.0001 | -14.97% |
| TC | -0.05(-0.08, -0.03) <0.0001 | -0.02(-0.03, -0.01) <0.0001 | -0.03(-0.06, -0.01) 0.0080 | 34.80% |
| TG | -0.05(-0.08, -0.03) <0.0001 | -0.00(-0.01, -0.00) 0.0320 | -0.05(-0.08, -0.03) <0.0001 | 6.01% |
| HDL | -0.05(-0.08, -0.03) <0.0001 | 0.00(-0.00, 0.01) 0.2480 | -0.05(-0.08, -0.03) <0.0001 | -3.40% |
| LDL | -0.05(-0.08, -0.03) <0.0001 | -0.00(-0.01, -0.00) 0.0480 | -0.05(-0.08, -0.02) 0.0020 | 8.60% |
| NHHR | -0.05(-0.08, -0.03) <0.0001 | -0.00(-0.01, -0.00) 0.0440 | -0.05(-0.07, -0.02) <0.0001 | 9.26% |

The adjusted variable is age(smooth), weight(smooth), height(smooth), BMI(smooth), history of past illnesses, history of hypertension, history of CHD, history of diabetes, history of previous surgeries, smoking and alcoholism.

Mediation analyses were performed using a probit-based causal mediation model. The reported mediation proportions were derived from the probit scale and should be interpreted with caution, particularly for a binary outcome. Greater emphasis should be placed on the direction and statistical significance of the indirect effects rather than the exact magnitude of the mediation proportions.

Abbreviations: PSM, propensity score matching; CI, confidence interval; WBC, white blood cell count; PLR, platelet-to-lymphocyte ratio; NLR, neutrophil-to-lymphocyte ratio; MLR, monocyte-to-lymphocyte ratio; SII, systemic immune-inflammation index; A/G, albumin-to-globulin ratio; TC, total cholesterol; TG, triglycerides; HDL, high-density lipoprotein cholesterol; LDL, low-density lipoprotein cholesterol; NHHR, non-HDL cholesterol to HDL cholesterol ratio.

Supplementary table 9. Association between albumin-corrected calcium and UL

| Exposure | Model I  OR (95%CI) Pvalue | Model II  OR (95%CI) Pvalue | Model III  OR (95%CI) Pvalue | Model IV  OR (95%CI) Pvalue |
| --- | --- | --- | --- | --- |
| Albumin-corrected calcium  (per 0.1 mmol/L increase, before PSM) | 0.54 (0.50, 0.58) <0.0001 | 0.54 (0.50, 0.58) <0.0001 | 0.54 (0.50, 0.58) <0.0001 | 0.53 (0.49, 0.58) <0.0001 |
| Albumin-corrected calcium  (per 0.1 mmol/L increase, after PSM) | 0.53 (0.48, 0.58) <0.0001 | 0.52 (0.48, 0.57) <0.0001 | 0.53 (0.48, 0.58) <0.0001 | 0.53 (0.48, 0.58) <0.0001 |

Model I no adjusted.

Model II adjusted for age(smooth), weight(smooth), height(smooth) and BMI(smooth).

Model III adjusted for history of past illnesses, history of hypertension, history of CHD, history of diabetes, history of previous surgeries, smoking and alcoholism.

Model IV adjusted for age(smooth), weight(smooth), height(smooth), BMI(smooth), history of past illnesses, history of hypertension, history of CHD, history of diabetes, history of previous surgeries, smoking and alcoholism.

Abbreviations: PSM, propensity score matching; UL, uterine leiomyoma; OR, odds ratio; CI, confidence interval.

Supplementary Figure 1. SMD and Probability Density after PSM

Abbreviations: PSM, propensity score matching; SMD, standardized mean difference.

Supplementary Figure 2. Nonlinear associations between inflammatory, lipid indicators and UL based on smoothing spline models before PSM

(A) Serum calcium. (B) WBC. (C) PLR. (D) MLR. (E) NLR. (F) SII. (G) Albumin. (H) Globulin. (I) A/G. (J) TC. (K) TG. (L) HDL. (M) LDL. (N) NHHR. The red solid lines represent the estimated effects, and the blue shaded areas indicate the 95% confidence intervals. The distribution of each variable is shown by the rug plot along the x-axis. All analyses were performed before PSM.

Abbreviations: PSM, propensity score matching; UL, uterine leiomyoma; WBC, white blood cell count; PLR, platelet-to-lymphocyte ratio; NLR, neutrophil-to-lymphocyte ratio; MLR, monocyte-to-lymphocyte ratio; SII, systemic immune-inflammation index; A/G, albumin-to-globulin ratio; TC, total cholesterol; TG, triglycerides; HDL, high-density lipoprotein cholesterol; LDL, low-density lipoprotein cholesterol; NHHR, non-HDL cholesterol to HDL cholesterol ratio.

Supplementary Figure 3. Mediating analysis of the correlation between blood calcium levels and UL using inflammatory and lipid indicators as mediators before PSM

(A) WBC. (B) PLR. (C) NLR. (D) MLR. (E) SII. (F) Albumin. (G) Globulin. (H) A/G. (I) TC. (J) TG. (K) HDL. (L) LDL. (M) NHHR. The total effect, direct effect, and indirect (mediation) effect are presented for each outcome. The proportion mediated indicates the percentage of the total effect explained by serum calcium. P values were estimated using nonparametric bootstrap methods.

Mediation analyses were performed using a probit-based causal mediation model. The reported mediation proportions were derived from the probit scale and should be interpreted with caution, particularly for a binary outcome. Greater emphasis should be placed on the direction and statistical significance of the indirect effects rather than the exact magnitude of the mediation proportions.

Abbreviations: PSM, propensity score matching; UL, uterine leiomyoma; WBC, white blood cell count; PLR, platelet-to-lymphocyte ratio; NLR, neutrophil-to-lymphocyte ratio; MLR, monocyte-to-lymphocyte ratio; SII, systemic immune-inflammation index; A/G, albumin-to-globulin ratio; TC, total cholesterol; TG, triglycerides; HDL, high-density lipoprotein cholesterol; LDL, low-density lipoprotein cholesterol; NHHR, non-HDL cholesterol to HDL cholesterol ratio.
